# Staff support in a National Health Service mental health trust in response to the COVID-19 pandemic: qualitative study

**DOI:** 10.1192/bjo.2022.12

**Published:** 2022-02-16

**Authors:** Holly Smith, Shuo Zhang, Abbeygail Jones, Sarah Dorrington, Helen Winter, Alison Beck

**Affiliations:** Corporate Psychology and Psychotherapy, South London and Maudsley NHS Foundation Trust, Maudsley Hospital, London, UK; Department of Child and Adolescent Psychiatry, Institute of Psychiatry, Psychology and Neuroscience, Kings College London, UK; and South London and Maudsley NHS Foundation Trust, Maudsley Hospital, London, UK; Corporate Psychology and Psychotherapy, South London and Maudsley NHS Foundation Trust, Maudsley Hospital, London, UK; Department of Psychological Medicine, Institute of Psychiatry, Psychology and Neuroscience, Kings College London, UK; and South London and Maudsley NHS Foundation Trust, Maudsley Hospital, London, UK; Corporate Psychology and Psychotherapy, South London and Maudsley NHS Foundation Trust, Maudsley Hospital, London, UK; Corporate Psychology and Psychotherapy, South London and Maudsley NHS Foundation Trust, Maudsley Hospital, London, UK

**Keywords:** Qualitative research, stigma and discrimination, policy, staff support, COVID-19

## Abstract

**Background:**

The COVID-19 pandemic has highlighted the impact work can have on healthcare workers and the importance of staff support services. Rapid guidance was published to encourage preventive and responsive support for healthcare workers.

**Aims:**

To understand mental healthcare staff's help-seeking behaviours and access to support at work in response to the COVID-19 pandemic, to inform iterative improvements to provision of staff support.

**Method:**

We conducted a formative appraisal of access to support and support needs of staff in a National Health Service mental health trust. This involved 11 semi-structured individual interviews using a topic guide. Five virtual staff forums were additional sources of data. Reflexive thematic analysis was used to identify key themes.

**Results:**

Peer-based, within-team support was highly valued and sought after. However, access to support was negatively affected by work pressures, physical distancing and perceived cultural barriers.

**Conclusions:**

Healthcare organisations need to help colleagues to support each other by facilitating open, diverse workplace cultures and providing easily accessible, safe and reflective spaces. Future research should evaluate support in the evolving work contexts imposed by COVID-19 to inform interventions that account for differences across healthcare workforces.

The COVID-19 pandemic has placed significant strain on healthcare services worldwide, and many healthcare workers have had to adapt to increased pressures and new responsibilities at work. In the UK, National Health Service (NHS) workers reported challenges such as staff shortages due to rules regarding self-isolation and difficulties accessing personal protective equipment (PPE).^1^ Making choices on allocation of constrained resources, and balancing patient care with their own needs, puts NHS staff at risk of sustaining ‘moral injury’.^2^ This is in addition to difficult experiences shared with the general population, such as social isolation, fear of infection and bereavement.^3^

The psychological impact of the pandemic on healthcare workers was widely acknowledged, resulting in UK-based NHS trusts making efforts to support staff through the development of new initiatives.^2^ Guidance emphasised the importance of both proactive and reactive support, sensitive to individual reactions to the pandemic.^4^ Evidence pointed towards the need to provide transparent information, enable adequate rest and facilitate peer support to protect healthcare workers’ mental health.^4^

At the South London and Maudsley NHS Foundation Trust (SLaM), staff were aware that the developing pandemic could exacerbate existing high rates of burnout and moral distress faced by mental health professionals.^5^ As non-clinical staff and many community teams moved to homeworking, formal support was made available virtually via the trust-based staff counselling service. Other existing support services delivered by the staff support team, including reflective Schwartz rounds attended by staff across the organisation and post-incident support sessions, moved online and were bolstered with additional structures. Five ‘rest and recharge’ hubs were set up across the trust for staff working on-site to get refreshments and take time away from working environments. Hubs were staffed by colleagues available to provide informal peer support and signpost to services and were a place where staff could pick up food and toiletry donations. A 24-hour advice and support line was created and self-help resources were disseminated. Trust board members and senior leaders began weekly live broadcasts to improve trust-wide communications. As the Black Lives Matter movement gained global momentum following the murder of George Floyd, a new staff forum ‘Time to Talk’ was introduced to facilitate discussion about race-based discrimination.

The intensity and acceleration of the pandemic demanded rapid changes to staff support provision in response to staff needs and evidence-based guidance. However, the specific needs of mental healthcare staff during the pandemic, and the impact the pandemic may have on access to staff support provision, were not known. To appraise staff support provision and inform ongoing initiatives, a qualitative project was conducted with staff working in a mental health trust. The aim was to understand help-seeking behaviours and access to support at work in response to the COVID-19 pandemic.

## Method

### Design and setting

All data collection was conducted as a formative process evaluation of ongoing staff support provision to inform iterative changes to this provision in the trust between June and August 2020. Qualitative methods were chosen to provide nuanced data on individuals’ subjective experiences of staff support services. This study was performed in accordance with the Declaration of Helsinki (1975), as revised in 2008. Ethics approval was not required as this was an evaluation of existing services, but consent was sought from interview respondents to record, transcribe and analyse their interviews, and for internal and external publication. Forum organisers agreed to the collection of anonymous and condensed notes. Consent was sought verbally and was recorded in project documentation saved securely on trust-based computer systems.

### Sampling and recruitment

Eleven staff involved in providing staff support during the pandemic were recruited through convenience sampling in July and August 2020. The rationale for recruiting this group was that in-depth data could be gathered from individuals with experiences of being both providers and recipients of support. Convenience sampling allowed for rapid data gathering and iterative changes to practice resulting from any implications. Members of the staff support team attended and observed staff forums and regular network meetings to collect a form of field notes. Data were gathered from both interviews and staff forums to strike a balance of ‘breadth and depth’: interviews allowed for follow-up questions and use of a topic guide to inquire directly about staff support provision, whereas forums attended by staff trust-wide enabled data to be collected from a range of individuals and workforces.

### Data collection

Individual interviews using a topic guide were conducted virtually by H.S. via Microsoft Teams. Interviews were recorded on MS Teams and transcribed verbatim by an external transcription company (Dictate2Us). The topic guide was developed to address key areas relating to COVID-19 work practices and staff support access such that it could inform changes to practice. Topics covered were: general well-being; COVID-19/infection control; ways of working; staff support; systemic issues; future thinking. Five virtual staff forums took place during the evaluation period and were observed as ‘naturally occurring focus groups’ attended by staff trust-wide. Three of these forums were organised as spaces for staff to discuss racial discrimination, one forum was organised as an opportunity for staff to provide feedback on remote working and the fifth was a reflective space about the impact of the COVID-19 pandemic. Demographic and workforce data were not collected from interview respondents to maintain their anonymity in internal trust-wide reports of the findings. Similarly, staff forums are considered ‘confidential’ spaces where attendees are not identified.

### Data analysis

Reflexive thematic analysis^6^ was used to analyse anonymised interview transcripts and forum notes. This method was chosen as it is an inductive approach allowing researchers to work within a phenomenological framework – finding meaning in the subjective experiences of respondents.^7^ This was important as the researchers worked in the same trust and had a mixed identity as researchers, providers and recipients of staff support services. Interview transcripts and notes from each forum were coded as a whole data-set. Themes that related to the same concept (e.g. ‘pressures on managers’, ‘challenges of home-based working’) were generated across cases (i.e. across all data). Initial codes were generated by H.S., then emerging themes were reviewed with S.Z. and S.D. as the coding scheme was refined and finalised.

## Results

Eighty-three basic themes were coded in total, grouped into 43 organising themes and 10 overarching global themes (see supplementary material available at https://doi.org/10.1192/bjo.2022.12). In this paper, we highlight themes with implications for staff support services across NHS providers.

### Team-based support

#### First port of call

When discussing help-seeking, team- and peer-based support was the most discussed source of support sought out by colleagues, regardless of workforce or seniority. Team-based support was strongly associated with the well-being of staff working remotely or on-site as front-line workers. The value of this team-based support was more its accessibility and immediacy than its content or eventual effectiveness:

‘For me what would be really interesting as well would be to know what kind of conversations staff were having with their line manager as well as each other […] I mean, that informal support is often where people will go first before they go anywhere else.’

In the natural focus groups, team-based support was spoken about more frequently than formal support, supporting the idea that informal support mechanisms are the primary target for help-seeking behaviours. Similarly, case-loads of formal support services mostly comprised staff experiencing interpersonal conflict within teams, suggesting that these services are sought when staff feel unable to rely on informal support:

‘… if they get what they need from that [informal support] … well, then they don't necessarily need to take that next step and do something that seems to be slightly more formal.’

#### Shared experiences

The context of COVID-19 heightened the value of peer support as colleagues faced new and unprecedented challenges. For example, colleagues on wards were asked to provide palliative care and community teams were asked to provide services remotely without knowing the potential impact on patients. Consequently, space and time to recognise the individual and collective impact of such experiences was highly sought after. Sharing experiences protected against feelings of isolation and engendered feelings of unanimity:

‘actually I think that peer support is really important as well because you're speaking to people who have an appreciation as to what you're going through at work because they're also going through it […] particularly in a ward donning PPE and knowing how that feels, knowing how it feels going onto a ward where you've got a COVID-positive or a potentially COVID-positive patient, where you might actually have to do end of life care with someone.’

### Access to formal support

Without devaluing the central role of informal support, some support providers felt that staff were unaware of formal support services available. Concerns were raised that a lack of clear pathways to formal support could limit engagement with new and existing services and discourage help-seeking:

‘the big challenge is are people aware of the options and, you know, what they could possibly tap into […] you know, because you could do as much as you like and you go and you speak to a team and you say something that you think everybody knows and then people say “Oh really? I didn't know about that”.’

Although team-based support may be ample for some, an over-reliance on it leaves colleagues without much-needed support. For instance, staff in need of help who are not comfortable sharing this with their team may be invisible to support services. This was exemplified by colleagues indicating that formal support is approached after team-based support options have been exhausted:

‘I'd often have people who come because they feel the responses are bad. I mean, I think that overall, the trust responds well, overall, it communicates well. There's a very good kind of global responses if that makes sense but I think that what's much harder is the individuals who fall through the gaps somehow …’

### Moderators of team- and peer-based support

#### Pressures on managers and capacity to support teams

Managers who were inflexible and inattentive to their teams’ needs during the pandemic were perceived negatively by staff in the natural focus groups. Experiences shared included being unable to book COVID-19 tests during working hours or being signposted too quickly to psychological well-being leaflets when raising concerns about the impact of changes to working practices, leaving staff feeling as though they had not been listened to. Variation in experiences of line manager support was thought to reflect differences in skill set and managerial style, with negative experiences making line managers less approachable for support:

‘To support somebody, you don't necessarily have to agree with everything they say, and you don't have to give them everything they want. That isn't really what support is, but it is about feeling listened to.’

‘I suppose it depends as well, just how good your line manager is, if you can manage to have those conversations […] because there's some excellent line managers out there who it's just part and parcel of what they do, and there's some who perhaps are not […] because it's just not in their skill set as such.’

Negatively perceived experiences of management may also be due to systemic pressures experienced across the organisation, which erode managers’ capacity to communicate empathically and create a compassionate working environment within their teams. In this environment, when even the most basic requests were denied, staff felt less able to seek help or express their struggles:

‘If I had a magic wand and I could create one change, it would be about teaching managers a different form of communication that was less defensive and more empathetic […] staff had talked for example about how people would get irritated when they took water breaks or they went to get food or whatever because everyone is stressed, and so that really filters down to the individuals.’

Endemic stress was described as a barrier to accessing support as pressures trickle down through organisational levels:

‘The organisation is stressed, which feeds down into the managers, who are stressed and they're given less empathy, and the managers are stressed and they give less empathy to the people under them.’

Line managers described feeling intense pressures as services moved to new working practices, and this had an impact on support given to staff. Middle management lacked access to necessary support and information as responsibilities were placed on them to provide equipment for home-based workers and coordinate COVID-19 risk assessments, leaving them feeling under-resourced and overwhelmed:

‘[…] watching [senior execs] and hearing them say “If you need office equipment, just ask your line manager”. But there not being any processes being made known to the line managers about how to actually get it done, I found that really difficult. I felt like they were setting us up to fail massively.’

#### Racism and discrimination

At forum events, staff from marginalised groups explained how experiences of racism and discrimination affected their access to support. The impact on their mental and physical well-being left them feeling exhausted, burnt out and unable to bring their ‘whole selves’ to work:

‘It's stress. Stress, having to fight, study harder to be promoted. All these things cause additional illness […] we're creating casualties […] because of the poor processes or the discrimination or the unconscious bias, whatever it is, we are actually creating patients from our staff.’

‘If staff are coming in to work but not bringing their whole self … How do you then try and manage your day-to-day task because you're performing, but not on full cylinders because you're not being you? So, that must be difficult that you've got to remember to split yourself so you can fit in in your place of work or try and assimilate.’

Staff members from marginalised groups described feeling unable to seek emotional support from White colleagues for such experiences, particularly after being on the receiving end of micro-aggressions. When help-seeking in teams, staff had experiences of being invalidated, made to feel it is ‘all in their head’ or being racially stereotyped as ‘difficult’ or ‘angry’. Consequently, staff from marginalised groups turned to formal channels of support, but this required a lot of courage, leaving many without any support at all:

‘If staff have an issue with patient care […] and they feel confident that they will get a fair hearing from whoever the line manager is, they will speak up and they will talk about it, because it's seen as being helpful to the process. If the experience of people is … and it might not be their experience, it might be their perceived experience that someone else has had in the team … is that they're not listened to or their concerns are dismissed then staff find it really difficult to speak up and they often won't […] They might use the freedom to speak up route but if it's your line manager and you then have to go above your line manager to get it sorted … [that's] a very brave thing to do.’

Tackling racism and discrimination was viewed as paramount to improving support provision. Managers were seen as playing a crucial role in this, reflected by staff from marginalised groups expressing how important it was that White colleagues in managerial positions were present in forums such as Time to Talk. Making existing cultural competency training mandatory was seen as a necessary first step, although some felt that the training in its current format is not sufficient:

‘My query would be how we're addressing the middle managers […] it's what are we doing at that level of management to make sure they're on board with staff support, and all of the changes that we need to make […] we can't eradicate racism but we can try and make it less impactful on staff.’

‘We've been asking for that [cultural competency] for a long time because why isn't it mandatory? […] but also that training doesn't even go far enough, it's very gentle […] people need to understand their own bias and how to counteract that bias because we're all people, we all have bias.’

#### Home-based working

Transitions to remote working led to concerns about team connectedness. Remote working was seen as reducing the accessibility of social and practical task-related support, leaving staff isolated and less able to share ideas with colleagues. Staff sought more immediate and informal access to their colleagues for support:

‘I think that's a big risk. And I know teams have their like morning zoning meeting or huddles and stuff but it's not the same. I think things can start feeling disconnected or people feel isolated not just literally or physically isolated working from home but just feeling less sense of belongingness or something like that, less part of a team.’

‘… you can't run something past a colleague just sitting in the office or get their thoughts or something so yeah, I'm not going to call somebody in Teams every two minutes and say what do you think so I felt myself making decisions on my own.’

Many teams organised online check-ins and social events to ‘stay connected’ with each other, further indicating shared concerns about social isolation and the value placed on team-based social support:

‘We set up regular tea breaks, so we have just … I think it's just half an hour here and there where we just take the laptop outside or we'll kind of have a cup of tea and just not to talk about work.’

‘I think the daily check-ins really helps. I think, you know, we didn't consciously acknowledge it was more of a support mechanism.’

## Discussion

Our formative evaluation highlighted the importance of team- and peer-based support among mental healthcare workers in the UK who had faced significant challenges since the COVID-19 pandemic began. It also indicated context-driven and systemic barriers to accessing such support, sign-posting the need for changes to staff support provision.

### Team-based support

The observed importance of informal support reflects recent studies involving health and social care professionals during COVID-19.^3^ Subsequent recommendations for supporting healthcare staff through COVID-19 include harnessing team- and peer-based support by upskilling supervisors to spot early signs of distress, ‘buddying up’ junior and experienced team members, and ensuring that structured peer support is available for staff, such as critical incident staff support.^4^ These recommendations are based on the principles of proximity, immediacy, expectancy and simplicity (PIES), which enable staff to continue working where possible and ensure that they are supported before their distress escalates into crisis. Informal support can also ‘de-medicalise’ distress by emphasising strengths-building and coping and moderating the impact of stigma on help-seeking for mental health issues.^5^ Research supporting the effectiveness of such interventions has typically been conducted on military populations.^8^ A rapid review of healthcare well-being interventions provides support for whole-systems and group-based interventions as preventive measures, although greater quality of evidence is desired.^9^

Existing interventions for staff well-being incorporate elements of peer support. For example, reflective practice groups are often embedded in clinical practice, enabling staff to discuss the emotional impact of their work and receive support within their teams.^10^ Similarly, Schwartz rounds allow staff to share emotional experiences with others from across the organisation and have been widely adopted by NHS trusts.^11^ One review of these interventions including 41 studies found that staff reported improved well-being and coping, job satisfaction, teamwork and empathy for colleagues as a result of these spaces.^11^ The continuation of such spaces therefore remains an integral part of staff support provision. At SLaM, findings in this evaluation led to ‘team well-being plans’ being developed and distributed as a means of encouraging collective problem-solving and to support team well-being in the longer term.

### Barriers to effective team-based support

Our findings indicate that pressures on managers, discrimination at work and remote working were perceived as key barriers to accessible and effective team-based support in the context of the emerging COVID-19 pandemic.

Organisational pressures can put strain on team leaders, which may cause them to be controlling or unsympathetic towards team members and be less likely to act with integrity or provide adequate support.^6^ Staff well-being may therefore benefit from leaders being appropriately trained to support staff and themselves,^12^ particularly in the contexts of organisational change or services under pressure during COVID-19. Team-leader training is now being more widely integrated into the staff support provision of SLaM, aiming to improve leaders’ understanding of burnout and workplace stress and help them to look after themselves. Additionally, the sessions aim to upskill managers to have conversations about well-being using active listening skills and to improve their awareness of available support services, which can be disseminated to colleagues.^13^

The experiences of racism and discrimination reported in our findings, are known to add to workplace stress and to have an impact on confidence in workplace support mechanisms.^14^ Surveys of NHS staff have previously reported that staff from marginalised groups receive higher rates of abuse and bullying than White colleagues and perceive fewer opportunities for career progression.^15^ Employers need to go beyond listening and data gathering to provide evidence of action towards racial equality, for example working with human resources departments to change recruitment processes and the transparency of career progression pathways.^16^ Cultural competency training is currently implemented across NHS trusts, aiming to challenge prejudices towards marginalised groups by improving cultural awareness. However, cultural knowledge does not necessarily lead to a reduction in discriminatory behaviour, and such training may reinforce negative beliefs and practices by homogenising marginalised groups.^17^ This may point to the need for organisations to adopt novel approaches that advocate ongoing individual reflexivity on personal beliefs and prejudices.^18^ Teams may benefit especially from organisations providing such training for team leaders, given the crucial role leaders have in supporting marginalised staff. Continuing to acknowledge the wider prevalence and impact of racial discrimination and changes to public narratives may also be important in supporting staff. There is an expectation of employers to be engaged with politics and take a stance on societal issues.^19^ Employees’ perceptions of corporate social responsibility, and organisational justice, are associated with employees’ sense of belongingness, meaningful work, job satisfaction and retention.^20,21^

There is currently a paucity of research into team-based support and well-being in remote-working teams, although concerns about social isolation and sense of belongingness in newly made remote teams have been acknowledged during the pandemic.^22^ Remote workers may benefit from using online platforms to replace informal conversations, allowing staff to discuss challenges, gain practical tips from co-workers and evaluate their performance after a difficult piece of work.^23^ Such platforms could be used alongside similar methods used by our participants to stay connected with colleagues to improve access to social and practical support in these teams. Further work is likely necessary to understand concerns about remote working now that this practice has become a ‘new normal’ for many and as lockdown restrictions ease, allowing individuals to see family and friends again and enabling more flexibility in working practices. Employers in the future may provide staff with greater choice regarding remote or on-site working,^24^ which could improve access to team-based support for those on-site or create new challenges for team cohesiveness. Research into this area will be particularly important as flexible and remote working become more commonplace in the NHS.

In addition to implications for support service delivery, the collection of data from existing staff forums illustrates a swift method of gathering qualitative data from large workforces. Given the time constraints and limited capacity of healthcare workers, existing meetings and forums can be used as sources of rich and nuanced qualitative data where repeated issues and challenges are discussed. In circumstances where information is needed promptly, accessing existing spaces is a mode of consultation not requiring an additional time commitment from participants.

### Strengths and limitations

Although many interview respondents were facilitators of support spaces, the majority were working remotely, which may have affected their ability to reflect on experiences of staff on-site. Additionally, knowledge of available support will have affected their responses. Our conclusions may therefore be unrepresentative of staff who ‘fall through the gaps’ and do not access workplace support. Future studies should look to recruit from more workforces with varying engagement with staff support. Data source triangulation from interviews and staff forums support the validity of findings.^25^ Our research team was made up of SLaM staff who therefore held ‘insider status’ and were keen for the findings to inform the iterative development of support offered to staff. This allowed focus group facilitators to have a good contextual understanding of the perspectives shared by staff, aligning with the interpretive phenomenological framework, but may have affected the approach to thematic analysis. S.Z. and S.D., whose roles are clinical and not directly involved in staff support provision within the trust, supported the confirmability and validity of findings through investigator triangulation.^26^ Pre-pandemic, there is evidence of higher workplace stress in mental healthcare compared with physical healthcare settings;^27^ however, it is likely that our findings also apply to staff in other healthcare settings, particularly where results reflect previous research.

## Data Availability

The data that support the findings of this study are not available because the transcripts contain information that could compromise the privacy of participants or other individuals.

## Supplementary material

For supplementary material accompanying this paper visit http://dx.doi.org/10.1192/bjo.2022.12. click here to view supplementary material
