# Supplementary material for: Staff support in a National Health Service mental health trust in response to the COVID-19 pandemic: qualitative study
Source: BJPsych Open. 2022 Feb 16;8(2):e49. doi: 10.1192/bjo.2022.12 (PMC8861548; doi:10.1192/bjo.2022.12)
Supplement: Supplementary file 1 [file S2056472422000126sup001.docx]

**Supplementary material**

Coding framework

| **Global theme** | **Organising theme** | **List of codes** |
| --- | --- | --- |
| COVID-19 | Lockdown | Lockdown |
|  |  | Lockdown |
|  | Risk assessment | Access |
|  | Self-isolation / shielding | Rule confusion |
|  | Testing | Access to swab test |
|  |  | Access to antibody test |
|  |  | NHS Test & Trace |
|  |  | Implication of results |
| Future thinking | Anxiety | Returning to work |
|  |  | 2nd wave |
|  | Forward planning | Charity |
|  |  | Beyond SLaM |
|  |  | Returning to work |
|  | New normal | What does it mean in practice? |
| Homelife | Childcare | Childcare concerns |
|  | Finances | Financial worries |
|  | Infection control | Infection control |
|  | Relationships | Relationships |
| Infection control | PPE | Confusion/uncertainty |
|  |  | Discomfort |
|  |  | Impact on work |
|  | Social distancing | At work |
| Mental health | Exhaustion | Burnout |
|  |  | Fatigue |
|  |  | Burnout |
|  | Loss and bereavement | Loss and bereavement |
|  | Sleep problems | Struggling with sleep |
|  | Social isolation | Loneliness |
|  | Stress and anxiety | Health anxiety |
|  |  | High levels of stress |
| Service users | Community | More risk to hold |
|  |  | Guidance |
|  | Physical health/death | New territory |
|  | Wards | New rules |
|  |  | Higher acuity/challenging behaviour |
| Staff support / staff voices | Rest and recharge hubs | Appreciation of Rest and Recharge hubs |
|  |  | Use of Rest and Recharge hubs |
|  | Speaking up | Racism and discrimination |
|  |  | Barriers |
|  | Support for individuals | What is available? |
|  | Support for teams | Team leaders under pressure/ needing support |
|  |  | Peer support |
|  |  | RSR |
|  |  | Chaplaincy |
|  |  | Importance of feeling valued/cared for |
| Ways of working/staffing | Annual leave | When to take |
|  | Commute | Cycling |
|  |  | Driving |
|  |  | TfL |
|  | Redeployment | Anxiety/frustration |
|  |  | Team dynamics |
|  | Silver linings | Processes (e.g. reduced bureaucracy) |
|  |  | Perks (e.g. free food, parking) |
|  | Staffing | Pressure on managers |
|  |  | Heavy workloads |
|  |  | Short staffing |
|  |  | Shifts |
|  | Technology | Challenges |
|  | Working from home | Pros |
|  |  | Cons |
